# Distinct traces of mixed ancestry in western commercial pig genomes following gene flow from Chinese indigenous breeds

**DOI:** 10.3389/fgene.2022.1070783

**Published:** 2023-01-13

**Authors:** Yebo Peng, Martijn FL Derks, Martien AM Groenen, Yiqiang Zhao, Mirte Bosse

**Affiliations:** ^1^ State Key Laboratory of Agrobiotechnology, College of Biological Sciences, China Agricultural University, Beijing, China; ^2^ Animal Breeding and Genomics, Wageningen University & Research, Wageningen, Netherlands; ^3^ Topigs Norsvin Research Center, Beuningen, Netherlands; ^4^ Amsterdam Insitute of Life and Environment (A-Life), VU University Amsterdam, Amsterdam, Netherlands

**Keywords:** introgression, hybridization, selection, commercial pigs, gene flow

## Abstract

Studying gene flow between different livestock breeds will benefit the discovery of genes related to production traits and provide insight into human historical breeding. Chinese pigs have played an indispensable role in the breeding of Western commercial pigs. However, the differences in the timing and volume of the contribution of pigs from different Chinese regions to Western pigs are not yet apparent. In this paper, we combine the whole-genome sequencing data of 592 pigs from different studies and illustrate patterns of gene flow from Chinese pigs into Western commercial pigs. We describe introgression patterns from four distinct Chinese indigenous groups into five Western commercial groups. There were considerable differences in the number and length of the putative introgressed segments from Chinese pig groups that contributed to Western commercial pig breeds. The contribution of pigs from different Chinese geographical locations to a given western commercial breed varied more than that from a specific Chinese pig group to different Western commercial breeds, implying admixture within Europe after introgression. Within different Western commercial lines from the same breed, the introgression patterns from a given Chinese pig group seemed highly conserved, suggesting that introgression of Chinese pigs into Western commercial pig breeds mainly occurred at an early stage of breed formation. Finally, based on analyses of introgression signals, allele frequencies, and selection footprints, we identified a ∼2.65 Mb Chinese-derived haplotype under selection in Duroc pigs (CHR14: 95.68–98.33 Mb). Functional and phenotypic studies demonstrate that this *PRKG1* haplotype is related to backfat and loin depth in Duroc pigs. Overall, we demonstrate that the introgression history of domestic pigs is complex and that Western commercial pigs contain distinct traces of mixed ancestry, likely derived from various Chinese pig breeds.

## 1 Introduction

Introgression and hybridization played a distinct role in the evolutionary diversification of plants and animals (Dowling and Secor, 1997; Mallet, 2005; Arnold et al., 2008; Stukenbrock, 2016; Grant and Grant, 2019). Genetic material introgressed from sister lineages has often been adaptive in plant and animal evolution (Dowling et al., 2016; Burgarella et al., 2019; Janzen et al., 2019; Cao et al., 2021). In wild animals and plants, adaptive introgression played an essential role in disease resistance and environmental adaptation. Examples entail introgression in *P. trichocarpa* (Suarez-Gonzalez et al., 2016; Suarez-Gonzalez et al., 2018), *Zea mays* (Hufford et al., 2013), and sheep (Cao et al., 2021). Sometimes morphological characteristics changed, for example, wing patterning in *Heliconius* butterflies (Pardo-Diaz et al., 2012; Enciso-Romero et al., 2017). In modern humans, a variant of the *EPAS1* gene was introduced from Denisovans into Tibetans, which has proven beneficial to the adaptation of Tibetans to high altitudes (Huerta-Sanchez et al., 2014; Zhang W. et al., 2020). However, introgressed haplotypes can also have adverse effects. Examples are risk factors for type 2 diabetes, lupus, biliary cirrhosis (Sankararaman et al., 2014), and even COVID-19 inherited from Neanderthals (Zeberg and Pääbo, 2020).

Human activities have impacted over 75% of the global land area over the past ten thousand years (Venter et al., 2016; Bullock et al., 2018). Domestication and dispersal of pets, plants, and livestock have strongly altered the worldwide distribution of flora and fauna (Wichmann et al., 2009; Ottoni et al., 2013; Koch et al., 2015; Bullock et al., 2018). During the first industrial revolution, humans deliberately promoted crossbreeding of local animal and plant breeds to accelerate the process of breeding. Human-mediated hybridization between different breeds has been an important factor in shaping domestic plants and animals’ genomic and phenotypic diversity (Larson and Burger, 2013; Meng et al., 2018). The hybridization from bovine ancestors improved Mongolian yak management and breeding (Medugorac et al., 2017). Likewise, haplotypes introgressed from Holstein and Brown Swiss affect protein and fat content of milk, calving traits, body conformation, feed efficiency, carcass, and fertility traits (Zhang et al., 2018).

Pigs have a long history of admixture. In the genus *Sus*, post-divergence interspecific admixture occurred before the domestication of *Sus scrofa* (Frantz L. A. F. et al., 2013; Frantz et al., 2014; Frantz et al., 2016; Liu et al., 2019). For *Sus. scrofa*, *Sus. cebifrons*, and *Sus. verrucosus*, around 23% of their genomes have been affected by admixture during the later Pleistocene climatic transition (Frantz et al., 2014). Gene flow also happened extensively between domesticated pigs to their wild ancestors during the domestication process (Giuffra et al., 2000; Frantz A. C. et al., 2013; Zhu et al., 2017). Hybridization between China and Western animals may date back to the 1st—fourth century AD (Wang et al., 2011). Historical records report that Chinese pigs were repeatedly introduced into Europe to improve the local pig breeds from the 18th century (Giuffra et al., 2000; Wang et al., 2011; White, 2011), followed by introduction into America from the 19th century onwards (Wang et al., 2011; White, 2011). Vice versa, Western commercial pigs were introduced into China since the start of the 20th century (Wang et al., 2011; White, 2011). The complex hybrid history between China and Western pigs has shaped the present genomic landscape in pigs.

There are 118 native pig breeds in China (Megens et al., 2007) with diverse phenotypic characteristics. Characteristic for Eastern Chinese pigs is early sexual maturity, higher ovulation number, and higher litters size (>15 for some breeds) (Wang et al., 2011). South Chinese pigs have inferior reproductive performance (8–10 piglets per parity for Luchan pigs), thinner skin, and excellent heat resistance (Wang et al., 2011; Chen et al., 2020).

In recent years, genomic studies have revealed some Chinese haplotypes in Western pig breeds that were likely introgressed and selected for. *AHR* is a toxicity- and fertility-related gene (Denison et al., 2011; Onteru et al., 2012). It is introgressed from a Chinese breed into Dutch Large White pigs (Bosse et al., 2014). Based on the Illumina Porcine 60 K SNP Beadchip dataset of Erhualian, White Duroc × Erhualian F2 population, Duroc and Landrace pigs. Yang et al. found a mutation in *VRTN* that increased vertebra number, carcass length, and teat number in Western pigs and was inherited from Chinese Erhualian pigs (Yang et al., 2016). The meat quality-related genes (*SAL1*, *ME1*) and fertility-related genes (*GNRHR*, *GNRH1*), are reported as being introgressed into Duroc from Meishan pigs by Zhao et al., using whole-genome re-sequencing data of 32 Chinese Meishan and 31 Duroc pigs (Zhao et al., 2018). Recently, Chen et al. also explored whole-genome sequencing data from 266 Eurasian wild boars and domestic pigs. They found that the *GOLM1-NAA35*, a gene that is responsible for cytokine interleukin 6 (IL-6) production in human immune cells (Li et al., 2016), is inherited from south Chinese pigs (SCN) in French Large White (LWHFR) (Chen et al., 2020). They also found a haplotype spanning *KATNAL1* that originated from east Chinese pigs (ECN) pigs and has been selected to increase the fertility in LWHFR pigs. Although only LWHFR and two Chinese native pig groups were included, their study provided the novel perspective that introgression from Chinese pigs to commercial breeds may vary considerably. Therefore, in the current study we extensively explore source of introgression and genomic regions that contained introgressed segments on a large scale, including multiple Western pig breeds and a broad sampling of Asian breeds.

Thus, many genomic segments from local Chinese pigs that contributed to favorable characteristics of Western commercial breeds have been identified (Bosse et al., 2014; Frantz et al., 2015; Chen et al., 2017; Chen et al., 2020; Wang et al., 2020), but these records are sporadic, and no systematic survey has been conducted. How extensive these episodes of introgression and improvement of Western domesticated pigs with animals from Asia have been, and where in China these pigs originated, are still unanswered questions. Although Chinese pigs are highly polymorphic (Amaral et al., 2008; Frantz A. C. et al., 2013; Zhao et al., 2019), they form a close genetic group, and there has been an extensive genetic exchange between (local) breeds (Huang et al., 2020). Disentangling the sources of the introgressed haplotypes will shed new light on historical breeding practices, help understand the molecular mechanisms underlying phenotype change, and be of great significance to future breeding.

Even though the overall level of introgression from Chinese pigs to Western commercial breeds seems relatively stable across breeds, the underlying haplotypes, genomic loci, and breed origins may vary (Bosse et al., 2014). In this paper, we present a comprehensive study of the gene flow of pigs from different Chinese origins into five distinct Western commercial lines and illustrate the difference of global haplotype introgression patterns between donor-recipient combinations.

## 2 Materials and methods

### 2.1 SNP calling, phasing, and imputation

The datasets analyzed during the current study are available from the NCBI Sequence Read Archive (http://www.ncbi.nlm.nih.gov/sra/) under project PRJEB1683 (Groenen et al., 2012), PRJEB29465 (Grahofer et al., 2019), PRJEB9922 (Frantz et al., 2015), PRJNA186497 (Li et al., 2013), PRJNA213179 (Ai et al., 2015), PRJNA231897, PRJNA238851 (Wang et al., 2015), PRJNA254936, PRJNA255085 (Ramirez et al., 2015), PRJNA260763 (Choi et al., 2015), PRJNA273907, PRJNA305081, PRJNA305975, PRJNA309108 (Li et al., 2017), PRJNA314580, PRJNA320525 (Bianco et al., 2015), PRJNA320526, PRJNA320527, PRJNA322309, PRJNA369600, PRJNA378496 (Zhao et al., 2018), PRJNA398176 (Zhu et al., 2017), PRJNA438040, PRJNA488327 (Yan et al., 2018), PRJNA488960 (Zhang Y. et al., 2020), PRJNA524263 (Zhang W. et al., 2020), and PRJNA550237 (Chen et al., 2020).

A total of 730 samples were included with Asian, Western, domestic and wild backgrounds (See Table S1). Raw reads were aligned to the Sscrofa11.1 reference genome (Warr et al., 2020) using the bwa-mem algorithm (Li and Durbin, 2009). Samtools-v1.8 (Li, 2011) was used for sorting, merging, and marking potential PCR duplications. Finally, haplotype-based variant detection was conducted with freeBayes-v1.1 (--min-base-quality 10 --min-mapping-quality 20 --min-alternate-fraction 0.2 --haplotype-length 0 --pooled-continuous--ploidy 2 --min-alternate-count 2) (Garrison and Marth, 2012). After SNP calling, SNP loci were screened and retaining with a quality value greater than 20 (vcffilter -f “QUAL> 20”). Further quality control was conducted with the following criteria: minor allele frequency (MAF) > 0.01, missing rate <0.01, call rate >90%, sequencing depth of sample >4. Individuals and loci satisfying the above criteria were retained for futher analyses, and assigned to their specific background (193 Chinese indigenous pigs, 30 Asian wild boars, 13 Yucatan mini-pigs, 40 Western wild boars, 298 Western commercial pigs and 18 wild suidae; Supplementary Table S1). Finally, phasing and imputation were performed based on this data set with Beagle 5.1 (Browning et al., 2018; Browning et al., 2021) (window = 20 overlap = 4 gp = true ap = true).

### 2.2 Genetic structure analysis

T-SNE dimensionality reduction was first conducted by *sklearn. manifold.TSNE* (*n_components* = 2, *perplexity* = 24) in scikit-learn-0.23.1 python package on high-quality *Sus. Scrofa* samples. To construct the Neighbor-joining tree (NJ-tree), we calculated the IBS distance matrix by plink-1.9 on phased SNP data with default parameters. Then the NJ-tree was constructed by fastME-v2.15 (-D 1 -m N -b 10000 -T 10 -s -I) (Lefort et al., 2015) with *Sus cebifrons* as the outgroup. The tree was plotted by the iTOl-v5 online tool (Letunic and Bork, 2021). Model-based global ancestry estimation was conducted with Admixture-1.3 (-B10 -c10) (Alexander et al., 2009) with cross-validation to assess the best fitting K-value.

### 2.3 Local introgression detection

Western wild boars and Yucatan minipigs were combined as the Western haplotypes background for the introgression study. Chinese groups were set as the donor population for every commercial line. Then putative introgression segments were detected for every donor-recipient combination with relative Identity-by-descent (rIBD) method using whole-genome sequencing data (Bosse et al., 2014). Identity-by-descent (IBD) detection was performed with the refinedIBD algorithm (length = 0.1 trim = 0.01 lod = 1) (Browning and Browning, 2013). These parameters were adjusted to detect not only segments that are identical, but segments with similar origins (i.e., Western or Asian) that show higher similarity than expected between Chinese and European ancestries.

The rIBD values were calculated on non-overlapping bins of 10 kb along the genome. For every bin, we calculated rIBD values with the following formula: $rIBD={nIBD}_{R,D}-{nIBD}_{R,B}$ . ${nIBD}_{R,D}$ denotes the normalized IBD (nIBD) value of the recipient-donor pair, ${nIBD}_{R,B}$ denotes the nIBD value of the recipient-background pair. $nIBD=\frac{Count\_IBD}{Total\_IBD}$ , Count_IBD = shared IBD counts between group1 and group2, ${Total\_IBD=N}_{1}{*N}_{2}$ . N_1_ and N_2_ are the sample size of group1 and group2, respectively. That way, rIBD >0 indicates that commercial breeds (recipient) shares more IBD traces with Chinese indigenous (donor) than Western background, and thus denotes introgression from the donor into the recipient population. In contrast, a negative rIBD value indicates that the number of haplotypes shared by the recipient and the background population is greater than that shared with the donor at that locus. We then performed a Z-transformation of the rIBD values with the mean and standard deviation values of overall IBD from all donor-recipient pairs. We independently selected the presumed introgression bins with a Z-rIBD threshold of μ+2σ for every pair, where *μ* and *σ* are the mean and standard deviation of Z-rIBD values. Positive significant Z-rIBD values are thus indicative of the presumed introgression from Chinese breeds into the Western commercial pig.

### 2.4 Overlapping ratio of Z-rIBD segments

To measure the coincidence of significant positive/negative Z-rIBD fragments between different donor recipients, we calculated the overlapping ratio by the following formula:

$${Overlapping\;ratio}_{C,A:B}=1-\left(\frac{{Noneoverlapped\;Counts}_{C,A}+{Noneoverlapped\;Counts}_{C,B}}{{Total\;Counts}_{C,A}+{Total\;Counts}_{C,B}}\right)$$

Where A, B, C denote the three populations. When we compare the overlapping level between “C - > A” and “C - > B”, population C denotes one donor population while A and B denote two different recipients. To compare the overlapping level between “A- > C” and “B- > C”, population C denote on recipient while A and B denote two different donors. ${Noneoverlapped\;Counts}_{C,A}$ : the number of positive/negative fragments/bins shared between C and A but not shared with B. ${Total\;Counts}_{C,A}$ : the total number of positive/negative fragments shared by C and A.

### 2.5 Selective sweep analysis

We performed a genome scan to detect recent adaptive introgression events using polymorphism data from the recipient populations only, using the VolcanoFinder-v.1.0 tool (Setter et al., 2020). The ancestral genome was constructed with 16-way Enredo-Pecan-Ortheu multiple alignments files, downloaded from the Ensembl v.103 databases (https://www.ensembl.org/). We obtained the allele frequency and the unnormalized site frequency spectrum files required for Volcanofinder, and performed the analysis according to the standard workflow from VolcanoFinder (https://doi.org/10.5061/dryad.7h44j0zr7) (Setter et al., 2020). Finally, variants were polarized by the ancestor alleles status and used as the input for VolcanoFinder-v1.0 (-big 30000, -1 1 1) (Setter et al., 2020).

### 2.6 Selection of introgression segments for further analysis

To locate important introgressed segments, we merged the consecutive significant ZrIBD bins into one introgression segment. We ranked introgression segments by segment length as the first criterion and average rIBD value as the second. Then, we selected the segments with a length larger than 11 Kb and Log (10) likelihood ratio of selective sweep footprint >11 (the 0.95 quantile). After that, we computed the length of the introgressed segment, selective sweep footprints, average ZrIBD value and average minor allele frequency for every introgression segment and selected the segments that matched all criteria as top candidates for further analysis.

### 2.7 Haplotype origin tracing

To trace the origin of haplotypes, alleles were first joined into a “FASTA” format sequence from the phased VCF file by an in-house python script. For a genomic region of interest, variants belonging to the same haplotype were joined to a sequence. These haplotypes thus consist of a string of variants derived from the phased VCF. Subsequently, the SNP distance matrix between haplotypes was calculated with SNP-dists v0.7.0 (https://github.com/tseemann/snp-dists). Finally, hierarchical clustering was conducted in R using the gplots package. Paterson’s D-statistics (Patterson et al., 2012) were computed by Dtrios (-j100) of the Dsuite v0.4 (Malinsky et al., 2021) tool package.

### 2.8 Determination of Chinese-derived alleles

We refer to an allele as a “Chinese-derived allele” when it occurs in Duroc and Chinese pigs, but is nearly absent in European wild boars. So the “Chinese-derived allele” should match the following criteria: 1) allele frequency in Duroc pigs≥0.1.2). Allele frequency in any of the Chinese local pig groups≥0.1.3) allele frequency in European wild boars≤0.0125 (i.e., only one European wild boar among 40 boars has that allele and is heterozygous).

### 2.9 Candidate variants selection and LD calculation

Chinese-derived variants were annotated by snpEff-v5.0 (Cingolani et al., 2012). To pinpoint potential causal variants with a high effect on the phenotype, the variants were then ranked using pCADD’s PHRED score. Briefly, the pCADD is the “pig combined annotation dependent depletion”, a model to score single nucleotide variants in pig genomes in terms of their putative deleteriousness, or effect on phenotypes, based on a combination of annotations, see (Gross et al., 2020). The pCADD model is a pig-specific variant of the original CADD model that was developed for human aimd aims to discriminate neutral variants from variants with high impact. Then, “Candidate variants” were selected by the following principles: 1) PHRED score >4.3 (the whole genome mean value). 2) missense variant, 3′UTR variant, or 5′UTR variant.

The LD level of the proxy SNPs with other variants from the sequence data was calculated by plink v1.90b6 (--ld-snp new14_97387849 --ld-window 3000 --ld-window-kb 3000 --r2 --ld-window-r2 0) in the Duroc population. The mean r2 values between proxy SNP and other variants in every block were used as the LD level of the proxy SNP and that block.

### 2.10 SNP selection from the illumina 50 K SNP array dataset

To be able to test phenotypic effects of the Chinese-derived introgressed haplotypes on chromosome 14, we wanted to expand our sample size by incorporating genotype data from commercial Duroc pigs. The genotype data was obtained from routinely screened pigs from Topigs Norsvin pigs that were genotyped by the (Illumina) Geneseek custom 50 K SNP chip with 50,689 SNPs (50 K) (Lincoln, NE, USA). The chromosomal positions are based on the *Sscrofa11.1* reference assembly. In our set of re-sequenced Duroc pigs, SNPs were filtered using the following requirements: Each marker had a MAF greater than 0.01, a call rate greater than 0.85, and an animal call rate >0.7. SNPs with a *p*-value below 1 × 10^−5^ for the Hardy-Weinberg equilibrium exact test were also discarded. All pre-processing steps were performed using plink v1.90b3.30 (Chang et al., 2015). SNPs on chromosome 14 were retained for further LD analyses to identify the SNP in highest LD with the candidate variants on the introgressed haplotypes. We tested LD between the candidate SNPs from the sequence in the Asian derived haplotype and SNPs on the 50 K chip by usingPlink-1.9 (--ld-snp new14_97387849 --ld-window 3000 --ld-window-kb 3000 --r2 --ld-window-r2 0).

### 2.11 Phenotype-genotype association

To estimate the impact of genotypes on production traits, we used the genotype data for the candidate SNP from 11,255 Duroc animals (not all animals have all phenotypes) to test the association of our introgressed allele with the following traits: daily gain from birth to Tstart (25 kg) for 9,921 animals, daily gain from Tstart to the Tend (25–120 kg) in 10,986 animals, backfat at 120 kg (Tend) in 7,192 animals, lean meat percentage and loin depth at the end (120 kg) in 7,688 and 7,192 animals respectively. The corrected phenotypes for all traits of each animal were obtained from the routine genetic evaluation by Topigs Norsvin. Then, for each trait, we conducted a Welch’s *t*-test (significance threshold *p* < 0.05) to test for differences in phenotypes of the different genotypes at our candidate SNP, that were assigned to either European or Asian background.

## 3 Results

### 3.1 Data collection

We collected 730 samples (Supplementary Table S1) from NCBI (https://www.ncbi.nlm.nih.gov/) and conducted SNP Calling with freeBayes-v1.1 (Garrison and Marth, 2012). After strict quality control, 19, 656, 271 SNPs and 592 samples were retained for further analyses, including 193 Chinese indigenous pigs, 30 Asian wild boars, 13 Western local pigs (Yucatan mini-pigs), 40 Western wild boars, 298 Western commercial pigs, and 18 samples from suidae in Southeast Asian islands (Supplementary Table S1).

### 3.2 Genetic structure analysis

**FIGURE 1:**
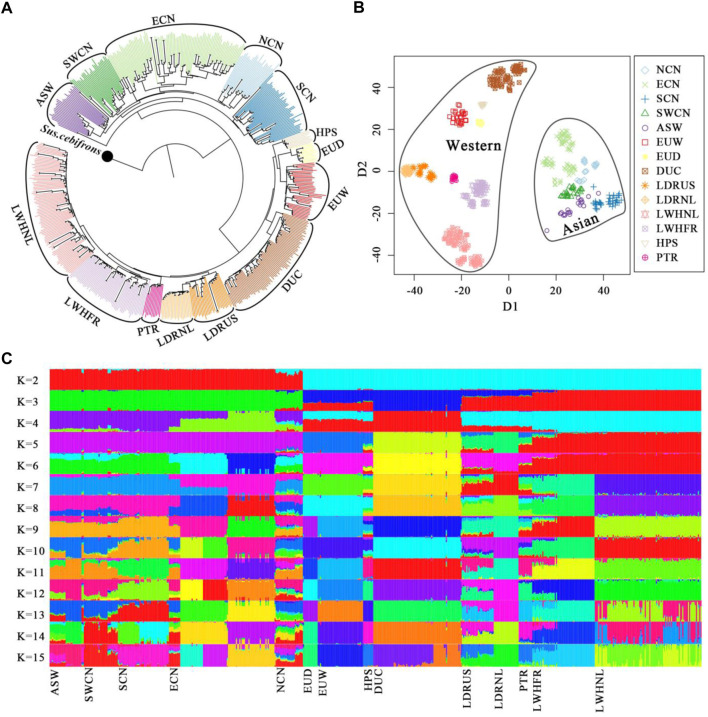
Genetic structure of pigs in this study. **(A)**. The Neighbor-joining tree was constructed by fastME-v2.1.5 based on the IBS-distance matrix and set *Sus. cebifrons* form Southeast Asian islands as the outgroup. **(B)**. Dimensionality reduction of whole-genome SNPs with the t-SNE algorithm. **(C)**. Global ancestry inference of Chinese and Western pigs conducted with ADMIXTURE-v1.3.0. NCN, North Chinese pigs; ECN, East Chinese pigs; SCN, South Chinese pigs; SWCN, Southwest Chinese pigs; ASW, Asian Wild boars; EUW, European Wild boars; EUD, European local pigs; DUC, Duroc pigs; LDRUS, American Landrace pigs; LDRNL, Dutch Landrace pigs; LWHNL, Dutch Large White pigs; LWHFR, French Large White pigs; HPS, Hampshire pigs; PTR, Pietrain pigs.

We predefined the groups of Chinese pigs according to our previous analysis (Peng et al., 2022) and their geographical origins (Wang et al., 2011) (Supplementary Figure S1). The NJ-tree, t-SNE dimensionality and global ancestry analysis were used to dissect the genetic structure of our samples. The NJ-tree and ancestry inference separate Western and Chinese-derived pigs (Figures 1A–C). Chinese animals clustered into a monophyletic clade, and different Chinese origins clustered into sub-clades except for Chinese Northern pigs (Figure 1A). For Western pigs, every commercial line clustered into a monophyletic clade (Figure 1A) and breeds were clearly distinguished in the t-SNE plot (Figure 1B). Admixture analysis was consistent with this pattern, with increasing values of K above six indicating local ancestry for European pigs, and Asian substructure was best captured with K = 14, when the cross-validation reached a plateau (Figure 1C and Supplementary Figure S2). After we removed a few of the eastern Chinese samples that showed hybrid ancestral components in the admixture result, we assigned the China and Western pig breeds to specific clusters according to geographical sources and genetic relationships.

Finally, Duroc (DUC), Dutch Large White (LWHNL), French Large White (LWHFR), Dutch Landrace (LDRNL), and American Landrace (LDRUS) were recognized as five distinct Western commercial lines (WS). European wild boars (EUW) plus local European pigs (EUD) were defined as the Western background population (WB). Moreover, Southern (SCN), Eastern (ECN), Northern (NCN), and Southwestern (SWCN) Chinese pigs were defined as the four Chinese local groups (AB). We combined Chinese wild boars (CNW), Korean wild boars (KRW), and Thai wild boars (THW) into the Chinese background population (AB) (Supplementary Table S1).

### 3.3 Introgression landscape from Chinese to western pigs

**FIGURE 2:**
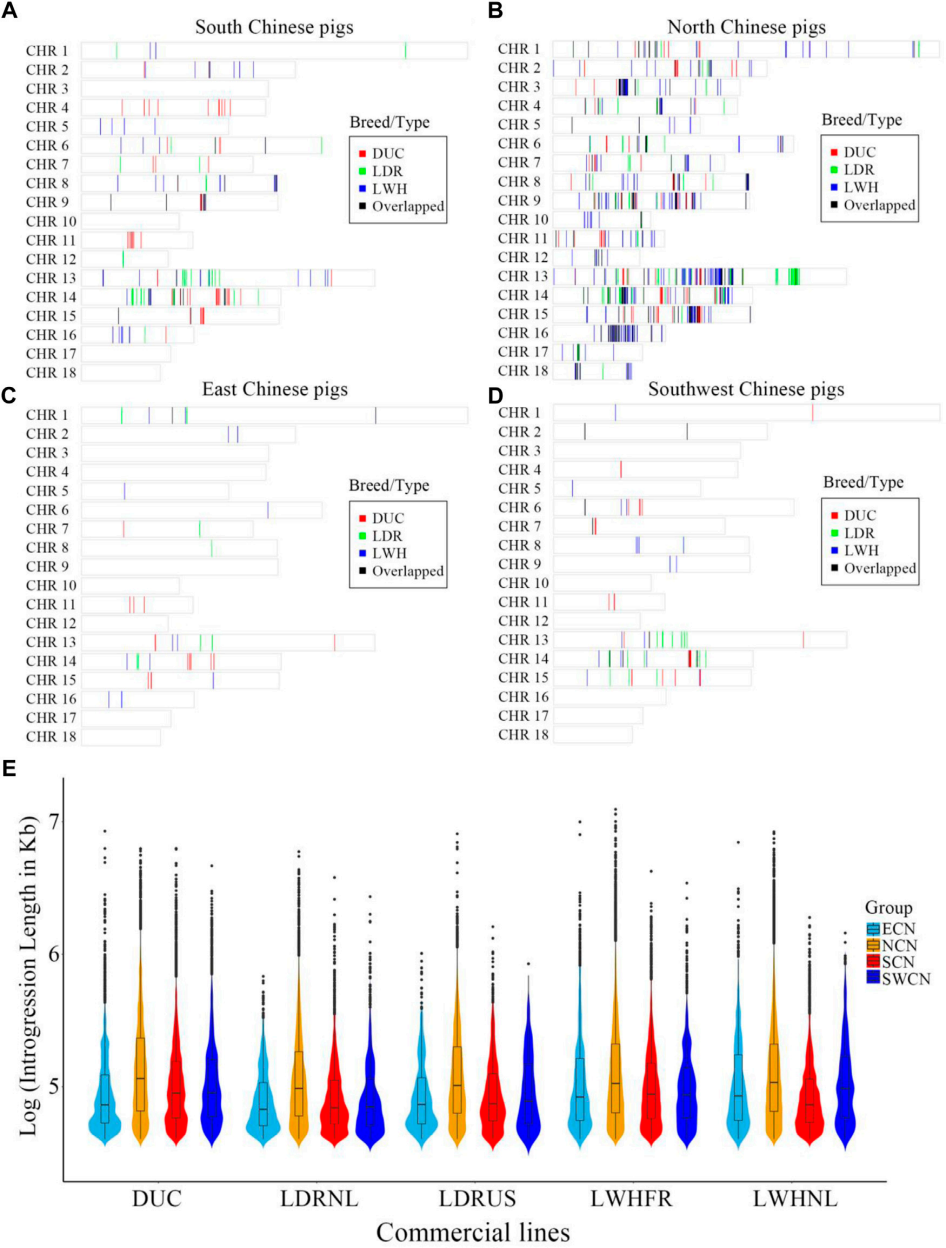
The distribution of genomic regions with introgression signature from **(A)** South Chinese pigs, **(B)** North Chinese pigs, **(C)** East Chinese pigs, and **(D)** Southwest Chinese pigs to different Western commercial breeds. DUC: Duroc, LDR: American and Dutch Landrace pigs, LWH: French and Dutch Large White pigs, Overlapped: the overlapped introgressed region between any two pairs. **(E)**. The features of natural logarithms transformed introgressed fragment lengths (in Kb) from China to Western pigs. ECN, East Chinese pigs; NCN, North Chinese pigs; SCN, South Chinese pigs; SWCN, Southwest Chinese pigs. DUC, Duroc; LDRUS, American Landrace pigs; LDRNL, Dutch Landrace pigs; LWHFR, French Large White pigs; LWHNL, Dutch Large White pigs.

**FIGURE 3:**
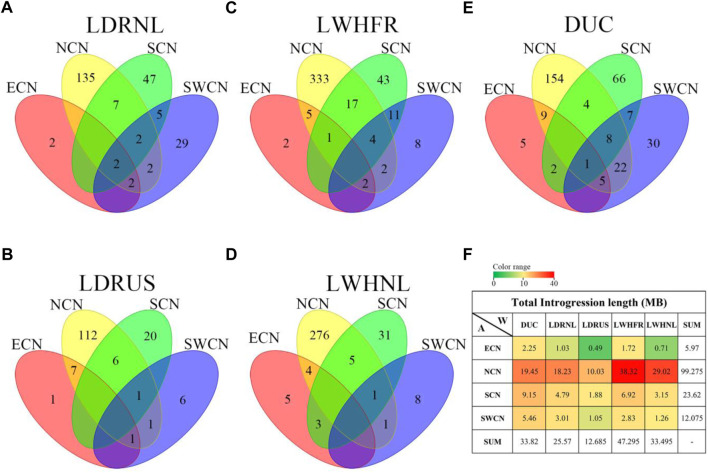
Venn diagram of gene counts on the putative introgression fragments from Chinese groups to Western commercial breed lines. **(A)**. LDRNL as the recipient. **(B)**. LWHFR as the recipient. **(C)**. DUC as the recipient. **(D)**. LDRUS as the recipient. **(E)**. LWHNL as the recipient. **(F)**. Total length (in Mb) of putative introgression segments (The overlapped introgression has been masked in the “SUM” column and row.).

We assessed local signatures of introgression in the Western pig genomes using an IBD haplotype sharing method. There are large introgressed fragments and introgression clusters from China to Western pigs (Figures 2A–D). On a genome-wide scale, the proportion and local regions of putative introgression are highly diverse between different donor-recipient pairs. The highest proportion of introgression into Western commercial breeds is NCN, followed by SCN (Figures 2A–D). The amount of introgression varied between the European breeds, with most putative introgression segments from Chinese pigs found in Large White breeds and the French Large White line in particular (Total length of Chinese-derived segments is 33.82 Mb for DUC, 25.57 Mb for LDRNL, 12.68 Mb for LDRUS, 47.29 Mb for LWHFR, 33.49 Mb for LWHNL. Figures 2A–D and Figure 3F). The putative introgression fragments also varied in length and number (Figures 2E, 3E). The longest introgression fragments reach ∼1.2 Mb between LWHFR and NCN (Total length: ∼38 Mb), but only ∼0.34 Mb between LDRNL and ECN (Total length: ∼1 Mb). For any Western commercial line, the average introgressed segment length from NCN is longer than from other Chinese populations (Figure 2E), suggesting a relatively recent genetic exchange between NCN and Western pigs.

We studied the number of genes affected by the introgression fragments for different donor-recipient pairs. Results are consistent with the total introgression length (Figure 3F). Most of the genes affected by introgression from local Chinese pigs into Western commercial pigs are specific for every donor-recipient pair (Figures 3A–E). Furthermore, most introgressed genes are from NCN to Large White (LWH), especially LWHFR (428 genes, Figure 3C).

### 3.4 Various segments from different Chinese groups are introgressed into specific western breeds

**FIGURE 4:**
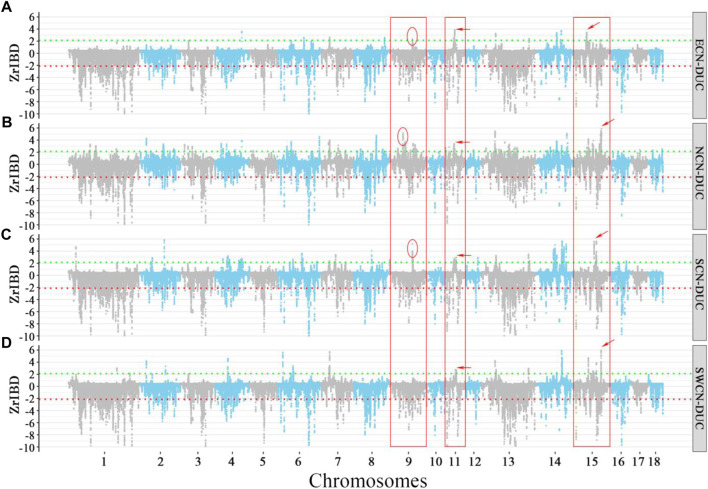
Manhattan plot of Z-rIBD values of Duroc *versus* different Chinese indigenous groups with European wild boars and Yucatan minipig as the background population. A positive Z-rIBD value indicates an introgression signal from a Chinese group to Duroc. In contrast, a negative value indicates Duroc shared more IBD fragments with a Western background population than the Chinese group (See method). Green and red dash lines are positive or negative significance levels (*mean ± 2sd*). **(A)**. The Z-rIBD dot plot with ECN as the donor. **(B)**. The Z-rIBD dot plot with NCN as the donor. **(C)**. The Z-rIBD dot plot with SCN as the donor. **(D)**. The Z-rIBD dot plot with SWCN as the donor.

We further compared the degree of overlap of positive/negative Z-rIBD segments among donor-recipient combinations to study the global introgression differences. We found that the overall positive Z-rIBD (introgression footprint, see method) patterns are less similar than negative Z-rIBD patterns in different donor-recipient pairs (Figure 4, And Supplementary Figure S3). For donor groups from different sources in China, the degree of overlap significant positive Z-rIBD segments between donor-recipient pairs range from 3.3% to 20.56% (Supplementary Table S2), but for negative Z-rIBD segments, it is 69.73%–93.51% (Supplementary Table S2). The degree of overlap of positive Z-rIBD segments is much lower than the negative Z-rIBD segments (Supplementary Table S2), suggesting specific introgression.

Here we take Duroc as an example. The difference in the positive peaks of Z-rIBD is noticeable (Figure 4). There are peaks at different locations or heights on chromosome nine for ECN, NCN, and SCN (Figures 4A–C), but there is no significant Z-rIBD peak for the SWCN-DUC pair (Figure 4D). On chromosome 11, there are peaks located at 34–39 Mb with different heights or widths (Figures 4A–D). Likewise, on chromosome 15, the highest peak is located at different positions for the Chinese groups (Figures 4A–D) except for NCN and SWCN (Figures 4B–D). Suggesting that pigs from different regions in China contributed differently to Western commercial pig breeds.

### 3.5 Hybridization occurred in the early breeding process of commercial pigs

**FIGURE 5:**
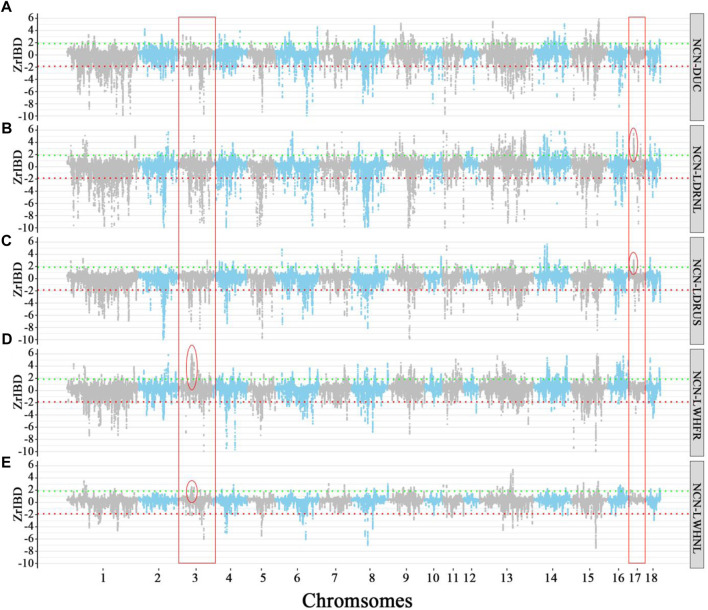
The Manhattan plot of Z-rIBD values of Northern China pigs *versus* Western pigs with European wild boars and Yucatan minipig as the background population. A positive Z-rIBD value indicates an introgression signal from a Chinese group to a Western commercial line. In contrast, a negative value indicates a Western commercial line shared more IBD fragments with the Western background population than a Chinese group (See method). Green and red dash lines are positive or negative significance levels (*mean ± 2sd*). **(A)**. The Z-rIBD dot plot with DUC as the recipient. **(B)**. The Z-rIBD dot plot with LDRNL as the recipient. **(C)**. The Z-rIBD dot plot with LDRUS as recipient. **(D)**. The Z-rIBD dot plot with LWHFR as the recipient. **(E)**. The Z-rIBD dot plot with LWHNL as the recipient.

There is a large difference in the introgression patterns between specific Chinese groups and Western commercial lines (Figure 5, And Supplementary Figure S4). The degree of overlap of positive Z-rIBD segments for the specific Chinese local pigs to different European commercial pigs ranges from 0% to 34.83% (Supplementary Table S3). In contrast, for the negative Z-rIBD segments, it ranges from 27.48% to 49.81% (Supplementary Table S3). However, the differences in the introgression patterns within related breeds from a specific Chinese group are smaller. Dutch and French Large White breeds show a similar introgression pattern from North Chinese pigs compared with other commercial pigs (Figures 5D,E, and Supplementary Table S3). The degree of overlap of positive Z-rIBD for these breeds is as high as 34.83% (Supplementary Table S3). In contrast, this is only around 10% compared to the other Western commercial lines (Supplementary Table S3). A broad peak on chromosome 3 (chr3:48–52 Mb) was found in Dutch Large White and French Large White (Figures 5D,E), but not in the other breeds (Figures 5A–C).

In the Landrace breed, the degree of overlap of positive Z-rIBD is as high as 26.55% (Figures 5B,C, and Supplementary Table S3). A significant introgression signal on chromosome 17 (CHR17:17–18 Mb) is observed in both Dutch and American Landrace (Figures 5B,C) but not seen in the other breeds (Figures 5A,D,E). Besides, the Z-rIBD peaks mentioned above are different in the different pig lines. The observed difference in introgression signal from specific Chinese groups to related Western commercial lines reflects a difference in the extent of introgression. This suggests that gene flow occurred mainly in the early stages of commercial pig breeding rather than after the differentiation of the lines. However, the tendency of artificial selection caused changes in signal strength.

### 3.6 A Chinese-derived haplotype introgressed into duroc genomes

**FIGURE 6:**
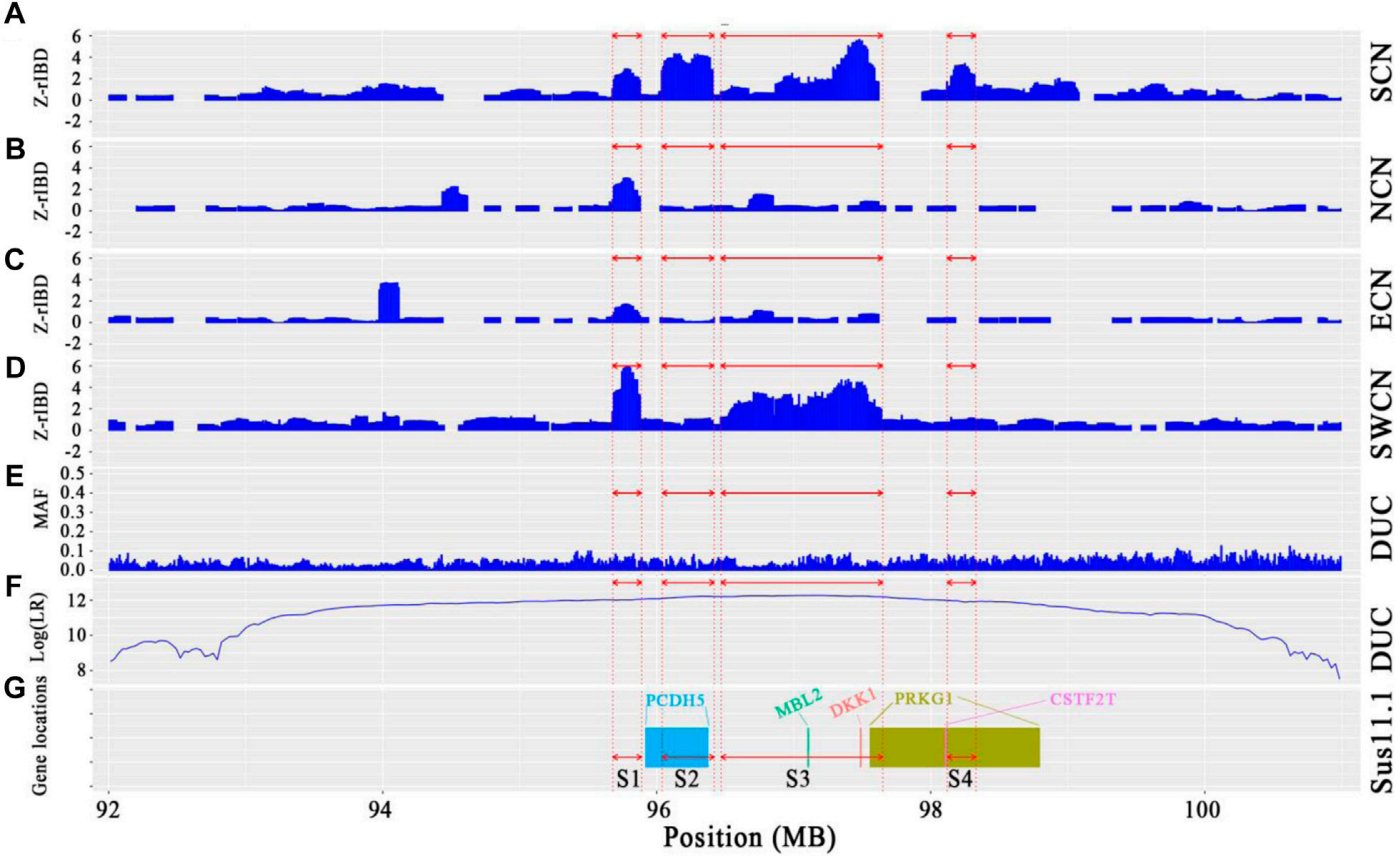
Local genome features on chr14:93.98–98.19 Mb of Duroc pigs. **(A–D)**. Z-rIBD values were calculated with Duroc as the recipient, European wild boars and Yucatan minipigs as the background together, and Chinese groups as the donor. **(E)**. Minor allele frequency of the Duroc population. **(F)**. Log-likelihood ratio of selection footprints calculated from VolcanoFinder-v1.0 tool. **(G)**. Candidate gene locations on *Sus. scrofa 11.1* reference genome. S1 denotes segment 1, which locate in chr14:95.68–95.89 Mb; S2 denotes segment 2, which locate in chr14:96.04–96.42 Mb; S3 denotes segment 3, which locate in chr14:96.47–97.65 Mb; S4 denotes segment 4, which locates in chr14:98.12–98.33 Mb.

We observed a cluster of Duroc-specific introgression signatures spanning ∼2.65 Mb on chromosome 14 (chr14: 95.68–98.33 Mb) (Figure 6). Such a strong introgression and selection signal is not seen for the other commercial pigs at that region (Supplementary Figures S5, S6). This introgressed region appears to be a set of segments derived from Chinese pigs in the Duroc population. The Z-rIBD value for SCN-DUC is up to 5.65 for segment 3 (the mean Z-rIBD value is 2.48 for segment 1, 3.90 for segment 2, 2.19 for segment 3 and 2.77 for segment 4, Figure 6A). Except for SWCN-DUC in segment 1 (mean Z-rIBD = 5.89, Figure 6D) and segment 3 (mean Z-rIBD = 4.74, Figure 6D), the mean Z-rIBD values is highest in the SCN-DUC pair (Figures 6A–D). We also observed a lower minor allele frequency than expected by chance (0.04 for this region but 0.12 for whole-genome) in Duroc (Figure 6E). These signatures are located within a strong adaptive selection region (chr14: 92–101 Mb) on the Duroc genome (Figure 6F). Moreover, there are five candidate genes in this region: *PCDH15*, *MBL2*, *DKK1*, *PRKG1*, and *CSTF2T* (Figure 6G).

Additionally, PCA plots of Duroc and Chinese pigs from SNPs across the full genome and local SNPs in this region display a strong discondancy (Supplementary Figure S7). The clustering of the Duroc and Chinese pigs in this region hint at introgression, evident from the big difference between the global and local PCA analyses. Combining the above results, we suspect that this haplotype in the Duroc genome was inherited from SCN or SWCN pigs.

**FIGURE 7:**
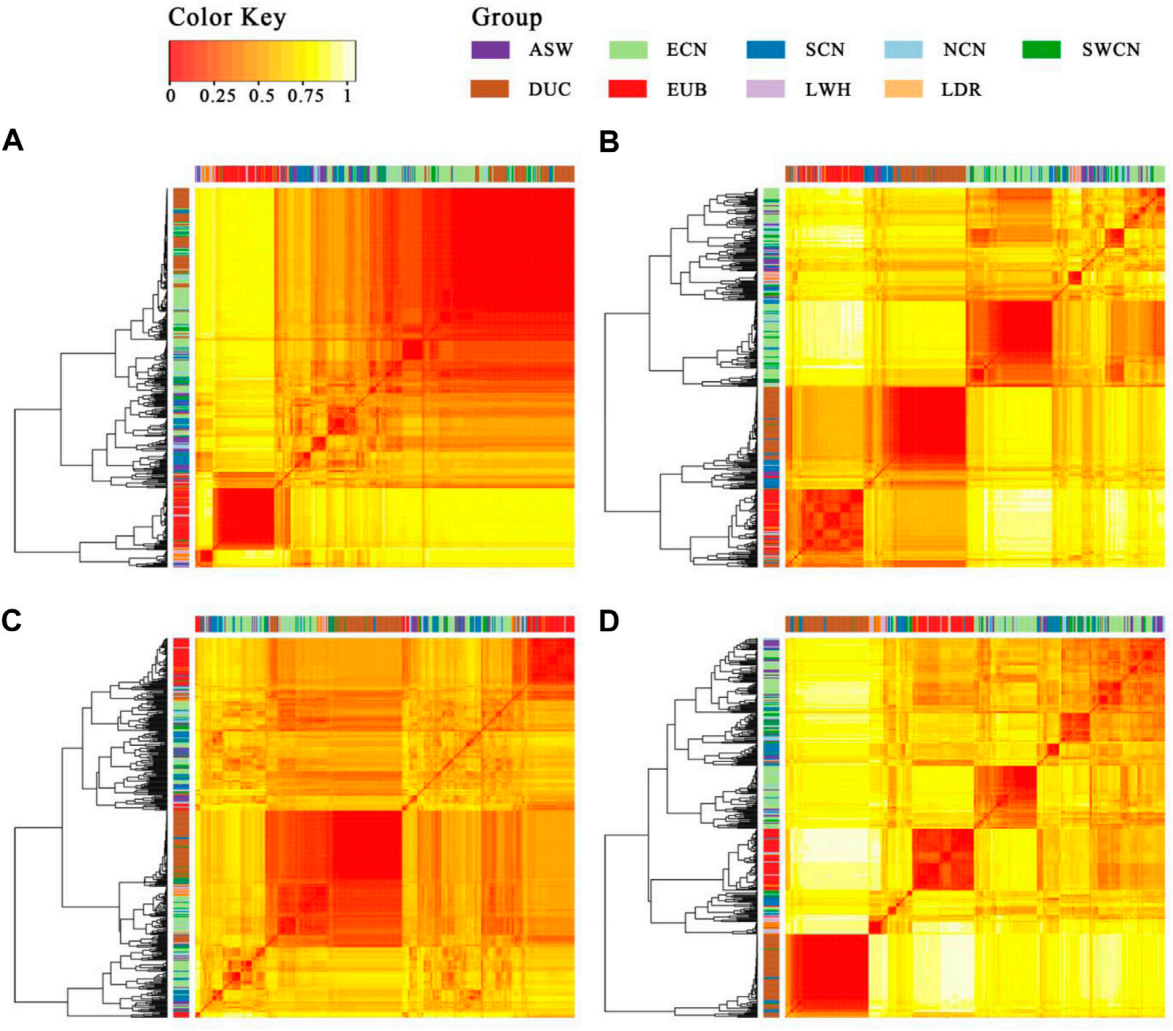
Heatmap and hierarchical clustering of the SNP distance matrix. **(A)**. Segment 1 (1343 SNPs were included); **(B)**. Segment 2 (1734 SNPs were included); **(C)**. Segment 3 (7210 SNPs were included); **(D)**. Segment 4 (1761 SNPs were included). SNP distance matrix was calculated with SNP-dists v0.7.0.

To trace the sources of the haplotype, we then calculated a distance matrix between individuals for every segment by SNP-dists v0.7.0 followed by hierarchical clustering in R-4.0 using the gplots package (Warnes, 2020). The results (Figure 7) show that most Duroc pigs clustered together with Chinese pigs (especially with ECN, SCN, and SWCN), in sharp contrast to LWH and Landrace (LDR). The LWH and LDR clustered with Western background populations on segment 1 and segment 4 (Figures 7A,D). In the clustering of segments 2 and 4, more SCN pigs are located witnin Duroc clades (Figures 7B,D), suggesting that fragment 2 is more likely derived from SCN pigs. The results of the ABBA-BABA test (D-statistics) highlights that Duroc shares more derived alleles with SCN than other Chinese pigs for segment 2, segment 3, and segment 4 (Table 1). Moreover, a high degree of linkage disequilibrium (LD) in this region (r2 = 0.56, Supplementary Figure S8) was found. According to the dist trees, D-statistics and the degree of LD, we believe that the haplotype (chr14: 95.68–98.33 Mb) in the Duroc genome is derived from SCN pigs.

**TABLE 1:** D-statistics result of four segments.

Segment	P1	P2	P3	D-statistic	Z-score	*p*-value
S1 (14:95.68–95.89 Mb)	EAS	DUC	EUW	0.322572	3.0973	0.001
	DUC	NCN	EUW	0.189092	2.7052	0.0034
	SCN	DUC	EUW	0.160682	1.1969	0.1157
	SWCN	DUC	EUW	0.158532	2.2859	0.0111
S2 (14:96.04–96.42 Mb)	EUW	DUC	ECN	0.132401	1.1937	0.1163
	DUC	EUW	NCN	0.0728634	0.8777	0.1901
	EUW	DUC	SCN	0.447761	6.6222	2E-11
	EUW	DUC	SWCN	0.187129	1.8819	0.0299
S3 (14:96.47–97.65 Mb)	ECN	DUC	EUW	0.266232	3.7459	9E-05
	DUC	NCN	EUW	0.0031214	0.0414	0.4835
	EUW	DUC	SCN	0.354104	9.3692	0
	SWCN	DUC	EUW	0.180207	2.7965	0.0026
S4 (14:98.12–98.33 Mb)	EUW	ECN	DUC	0.0861798	0.8438	0.1994
	EUW	NCN	DUC	0.0833274	0.9529	0.1703
	EUW	SCN	DUC	0.291517	3.3918	0.0003
	EUW	SWCN	DUC	0.114714	1.3727	0.0849

Note: S1, S2, S3, and S4 indicate segments 1–4 introgressed from Chinese pigs into commercial pigs. D=(ABBA-BABA)/(ABBA + BABA), with closely related *Sus* species from Southeast Asian islands as the outgroup. P1-P3, are the combination of EUW, and Chinese native pig groups (There are four valid combinations according to the formula of D-statistics).

### 3.7 Prioritizing causal variants within introgressed haplotypes

We further investigated SNP allele frequencies in the four segments in different pig populations (Table 2, And Supplementary Table S4). There are many alleles with low (≤0.0125) frequencies in Western wild boars but high frequencies in Duroc pigs for each of the segments (123 variants in segment 1,118 variants in segment 2,436 variants in segment 3,383 variants in segment 4, Supplementary Table S4). Furthermore, the derived alleles in Duroc pigs at these loci seem to have undergone strong selection (Figure 6F). We believe these are candidate alleles derived from Chinese pigs due to their moderate allele frequencies in Chinese pigs (Table 2, And Supplementary Table S4). Seven candidate mutations (Supplementary Table S5) were selected from the putative Chinese-derived set of alleles that potentially have a high functional impact (see methods). These variants are likely to have a strong impact on the phenotype as derived from the pCADD model, with the strongest located within the three prime UTR region of *PRKG1*.

**TABLE 2:** Average allele frequency of the Chinese-derived alleles within the four segments, in every population.

Segment	DUC	LDRNL	LDRUS	LWHFR	LWHNL	EUD	EUW	ECN	NCN	SCN	Secn	ASW
S1	0.8894	0.0022	0.0172	0.0264	0.0372	0.0003	0.0122	0.7238	0.5020	0.4289	0.7300	0.3062
S2	0.8827	0.0016	0.0058	0.0576	0.1149	0.0007	0.0123	0.1060	0.1123	0.4429	0.1874	0.2032
S3	0.8961	0.3528	0.3401	0.2664	0.2177	0.0004	0.0081	0.3453	0.3090	0.3500	0.5444	0.2241
S4	0.8818	0.0551	0.0602	0.0675	0.0448	0.0006	0.0046	0.1958	0.2003	0.3029	0.2138	0.1974

Note: S1, S2, S3, and S4 indicate segments 1–4 introgressed from Chinese pigs into commercial pigs. In the four segments, there are high allele frequencies in Duroc but low allele frequencies in Landrace and Large White, while these allele frequencies levels in Chinese pigs are high or moderate.

### 3.8 Association of *PRKG1-haplotype* with production traits

We analyzed genotype and phenotype data of 11,255 animals from a commercial Duroc population to assess the potential phenotypic impact of the introgressed haplotypes. We screened the (Illumina) Geneseek custom 50 K SNP array for SNPs in highest LD with the introgressed haplotypes, and a SNP (INRA0045978) was selected as a proxy for the introgressed segment due to its high LD (r2 range from 0.65 to 0.73) with the seven candidate alleles in the Duroc population (Supplementary Table S6, Supplementary Figure S9). Next, we used the genotypes for this selected SNP from 11,255 Duroc animals from the same commercial breed to test the association of INRA0045978 with a set of production traits (See methods).

**FIGURE 8:**
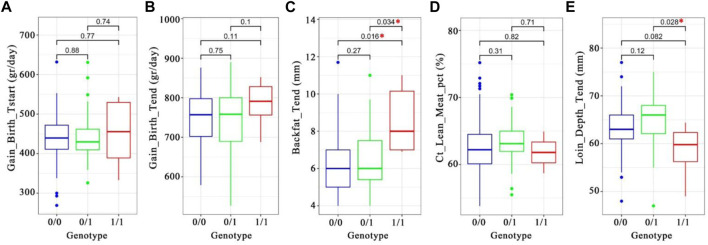
Box-plot of phenotype-genotype associations of the introgressed haplotype tagging SNP INRA0045978 (chr14:97387849) in ∼11,000 Duroc pigs. A–E, the t. test *p*-values were written on the plots. A star in red denotes significant difference between two genotypes. **(A)** daily gain from birth to starting (Grams per day). **(B)**. Daily gain from start to the end (Grams per day). **(C)**. Backfat at the end (Millimeters). **(D)**. Lean meat percentage (Percentage of lean meat). **(E)**. Loin depth at the end (millimeters).

We found a significant association with backfat (genotype “0/0” *versus* “1/1; *t*-test *p*-value 0.016; Figure 8C) with a and with loin depth (genotype “0/1” *versus* “1/1; *t*-test *p*-value 0.028; Figure 8E and Supplementary Table S7). The INRA0045978 SNP has a low Duroc reference allele frequency in Western wild boar (0.0125) but higher in Chinese pigs (0.5517) and Duroc (0.8782). These results suggest that the *PRKG1-haplotype* may decrease backfat (mean difference of 2.3 mm) and increase loin depth (mean difference of 6.1 mm) in Duroc pigs.

## 4 Discussion

We conducted a comprehensive analysis of the introgression from China to Western commercial pigs. The complexity of the commercial pig breeding process caused unforeseen scenarios. Our findings reveal the distribution and quantity of Chinese pig genetic components in major Western commercial pig breeds.

Interestingly, we found that the overall positive introgression patterns across breeds are less similar than negative patterns. The high degree of overlap for negative Z-rIBD segments was caused by the close genetic relationship among local Chinese pigs. The lower degree of overlap for the positive Z-rIBD segments indicates specific contributions from different Chinese local pigs into Western pigs. This could indicate that some genomic regions in Western pigs do not allow introgression from such distantly related pig populations and that purifying selection is at play. By contrast, breed-specific traits requirements could promote introgression reserved at specific loci, wherein other breeds, these Chinese-derived haplotypes, are undesired. Therefore, we hypothesize that genomic regions lacking Chinese introgression in all Western pigs contain genes that contribute to traits shared across all Western pigs and identify this as an exciting avenue for future research.

Introgressed sequences from different Chinese pig groups were found for a given Western breed. This may have been influenced by the opening of foreign trade ports in China hundreds of years ago and by the traits of pigs in different places (Chen et al., 2020). Western commercial breeds have retained different proportions and different specific loci of introgression. We believe different Chinese pig breeds were introduced for crossbreeding before current Western breeds were established. After establishing Western commercial breeds, these breeds were selected in different directions. We show introgression signals at the same genomic positions but with different introgression intensities for different lines from the same breed. This suggests the influence of directional selection on the gene flow. These results show that the variation in phenotypes of Western commercial breeds is caused by ⅰ) their initial variety, ⅱ) different Chinese pigs used for introgression, ⅲ) different directions and strength of selection after introgression. For different commercial lines of the same breed, the variation in phenotypes was most likely mainly caused by variation in the strength of selection. An illustration is the identified novel introgression haplotype from Southern China to Duroc pigs on chromosome 14 harboring the *PRKG1* gene. The *PRKG1* gene straddles the two introgressed segments (segment 3 and segment 4). Considering the high degree of LD in this region, it is very likely that they are derived from a single gene flow event. *PRKG1* has previously been reported to have undergone positive selection in Duroc (Kim et al., 2015) and is related to fatty acid composition. The gene showed copy number variation in Iberian - Landrace crosses (Revilla et al., 2017) and is related to average daily gain in Large White pigs (Wu et al., 2019). Furthermore, we showed that this introgressed *PRKG1*-haplotype significantly affects the thickness of the pig backfat and loin depth (Figures 8C–E), indicating its relevance for commercial breeding.

We also found other genes with essential functions in this region (Table 3). *PCDH15* is related to backfat thickness according to a GWAS result of Landrace and Yorkshire population (Lee and Shin, 2018). Porcine *MBL2* is one of the mannose-binding lectins; it is the central component of innate immunity, facilitating phagocytosis and inducing the lectin activation pathway of the complement system (Phatsara et al., 2007; Bergman et al., 2014). *DKK1* is one of the Wnt signaling inhibitors. Upregulation of *DKK1* expression can be observed in the endometrium in pigs during the pre-implantation period (Zeng et al., 2019). *CSTF2T* plays a potential role in infertility as a mutation in this gene caused male infertility in humans (Gorukmez and Gorukmez, 2020). In conclusion, the introgressed segment contains a set of genes with potential impact on backfat thickness, immunity, daily gain and reproduction.

**TABLE 3:** Genes overlapping with the four segments within the introgressed region.

Segment lable	Position (BP)	Name	Description
S1 & S2	chr14:95,920,700–96,372,532	PCDH15	Protocadherin related 15
S 3	chr14:97,103,926–97,107,635	MBL2	*Sus scrofa* mannose-binding lectin 2
S 3	chr14:97,487,117–97,490,450	DKK1	Dickkopf WNT Signaling Pathway Inhibitor 1
S 3 & S 4	chr14:97,558,535–98,793,356	PRKG1	Protein Kinase CGMP-Dependent 1
S 4	chr14:98,105,772–98,110,358	CSTF2T	Cleavage Stimulation Factor Subunit 2 Tau Variant

Note: S1, S2, S3, and S4 indicate segments 1–4 introgressed from Chinese pigs into commercial pigs. The name of the gene is GeneCards (https://www.genecards.org/) Symbol. Description information is from GeneCards.

We also observed a large number of introgressed haplotypes in commercial Western pig breeds derived from NCN. However, we did not find any relevant written records of such an introduction of NCN into Europe or America. A general view is that ECN/SCN has been introduced to Europe to improve Western commercial pig breeds (Chen et al., 2017; Zhao et al., 2018; Chen et al., 2020). We, therefore, assume that NCN did not participate in the crossbreeding with Western commercial pigs directly but that the haplotypes introgressed and retained in Western pigs are more conserved in NCN than SCN/ECN. This suggests that current NCN pigs resemble the local breeds introduced centuries ago. This assumption should, however, be confirmed in future studies. Furthermore, it is known that Western commercial pigs contributed to NCN after the 20th century. Ai et al. (2015) found an extreme divergence between the northern and southern Chinese pig haplotypes in the 14-Mb region on the X chromosome. These haplotypes found in NCN were also found in European pigs. Therefore, a reciprocal introgression from European-related boars to NCN and *vice versa* cannot be ruled out. Therefore, care should be taken when assessing the direction of selection and interpretation of the results.

## 5 Conclusion

A comprehensive analysis of the genetic introgression from Chinese pigs of different regions into different Western commercial lines was studied with 592 re-sequencing pigs. Our analysis revealed different Chinese pig haplotypes’ complex introgression patterns and characteristics into Western commercial pig breeds. The results showed that the amount and origin of haplotypes introgressed from different Chinese pig sources to specific Western pigs vary greatly. The impact of Chinese haplotypes from specific sources on different commercial breeds is very different. The introgression likely occurred in the early stages of breed development. Breeding selection tendency experienced by different lines likely led to the observed differences in gene introgression. LWH pigs are most affected by Chinese haplotypes and the haplotypes were better retained in LWHFR. We also found that a ∼2.65 Mb Chinese-derived haplotype in Duroc pigs significantly affects the thickness of the pig backfat and the increase of loin depth.

## Data Availability

The original contributions presented in the study are included in the article/Supplementary Material, further inquiries can be directed to the corresponding author.
