# Supplementary material for: Distinct traces of mixed ancestry in western commercial pig genomes following gene flow from Chinese indigenous breeds
Source: Front Genet. 2023 Jan 13;13:1070783. doi: 10.3389/fgene.2022.1070783 (PMC9880450; doi:10.3389/fgene.2022.1070783)
Supplement: Supplementary file 1 [file DataSheet1.docx]

## Supplementary figures:


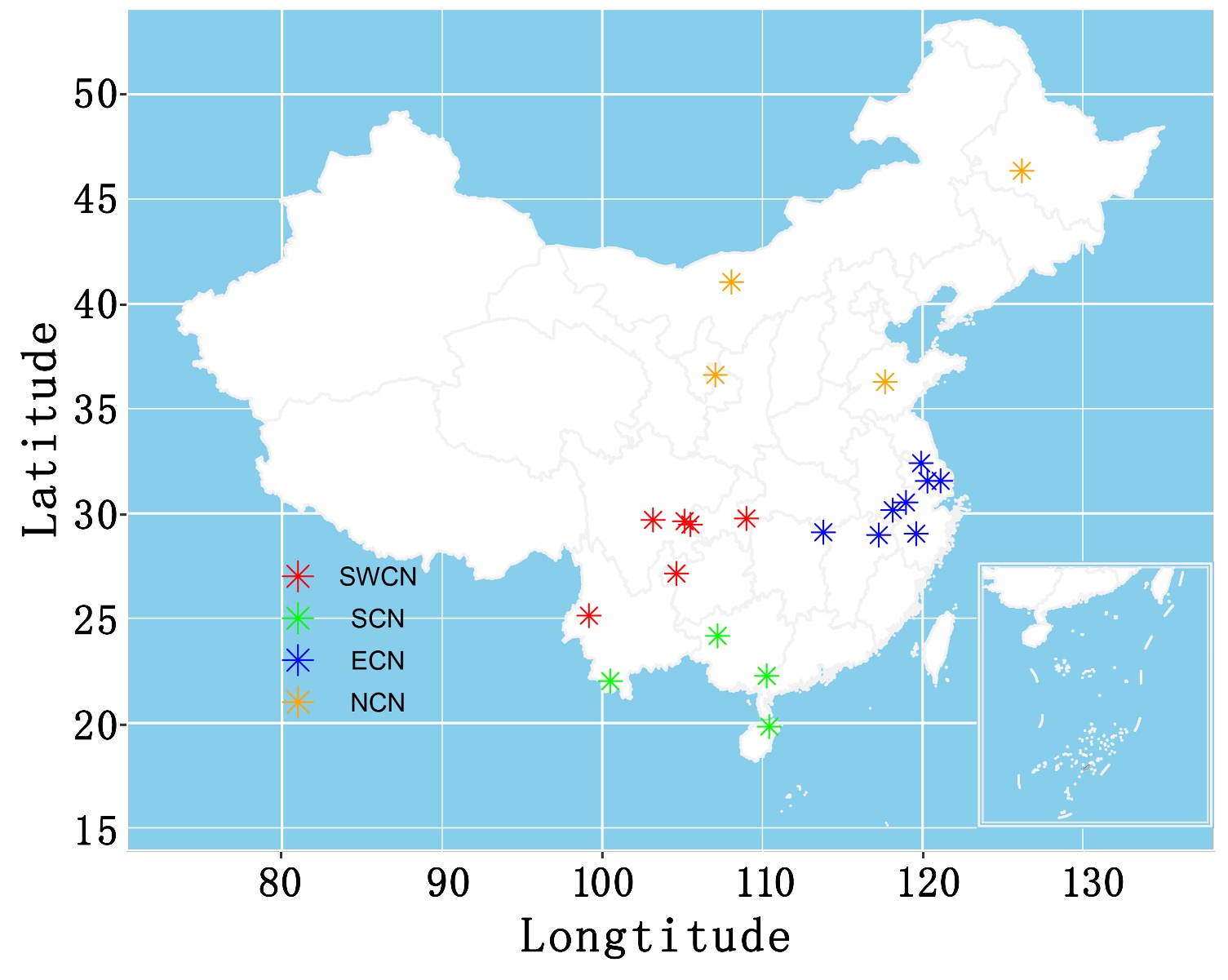


**Suppl. Fig. S1** The geographical location of Chinese indigenous pigs. The production centers (the city coordinates) of pig breeds were used as the geographical location of pigs (Wang., Wang. et al. 2011). SWCN: Southwestern Chinese pigs; SCN: Southern Chinese pigs; ECN: Eastern Chinese pigs; NCN: Northern Chinese pigs.


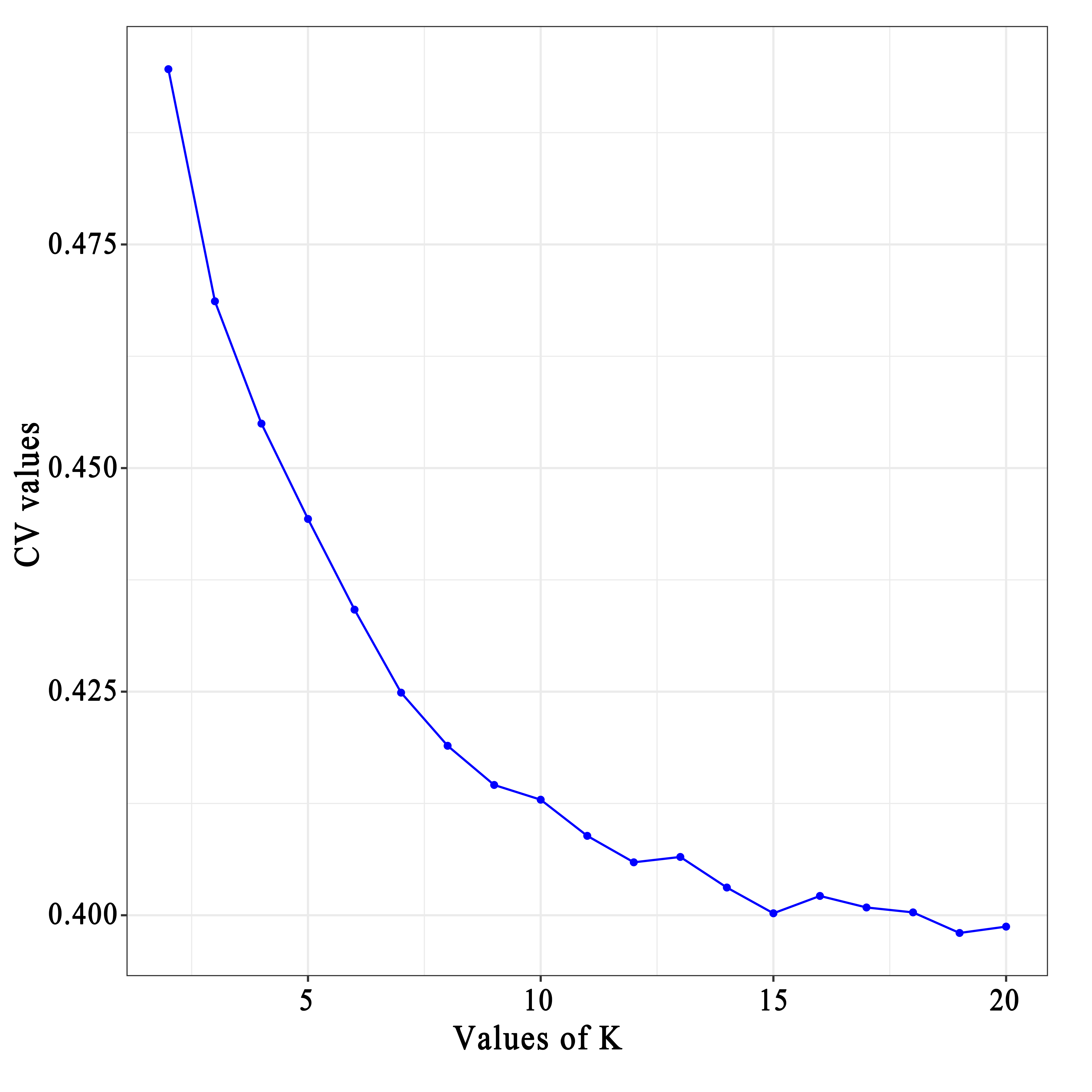


**Suppl. Fig. S2** Scree plot of CV values for k=2-20.





**Suppl. Fig. S3** Manhattan plot of Z-rIBD values from various Chinese indigenous groups versus specific Western commercial line with European wild boars and Yucatan minipig as the background population. (A). Dutch Landrace. (B). Dutch Large White. (C). American Landrace. (D). French Large White. A positive Z-rIBD value indicates an introgression signal from a Chinese group to Duroc, while a negative value indicates Duroc shared more IBD fragments with a Western background population than the Chinese group. Green and red dash lines are positive or negative significance levels (*mean±2sd*), respectively.





**Suppl. Fig. S4** Manhattan plot of Z-rIBD values of specific Chinese group versus different Western commercial groups with European wild boars and Yucatan minipig as the background population. (A). East Chinese pigs. (B). South Chinese pigs. (C). Southwest Chinese pigs. A positive Z-rIBD value indicates an introgression signal from a Chinese group to Duroc, while a negative value indicates Duroc shared more IBD fragments with a Western background population than the Chinese group. Green and red dash lines are positive or negative significance levels (*mean±2sd*), respectively.

**
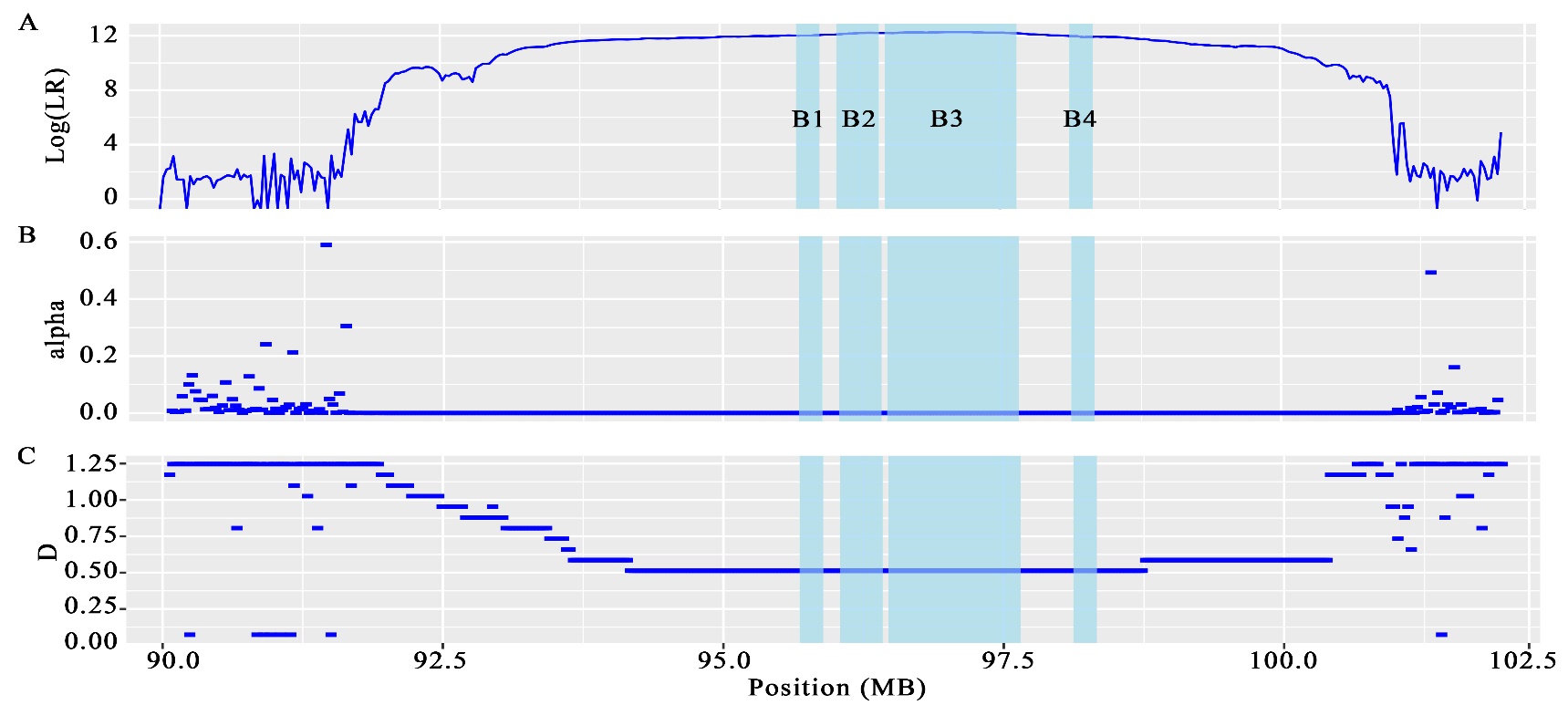
**

**Suppl. Fig. S5** VolcanoFinder result of Duroc pigs on chr14:92–102 MB. (A). Log likelihood ratio of the selection footprint. (B). Selective strength $\alpha$. (C). Predicted divergence between the target and the ancestor species.

**
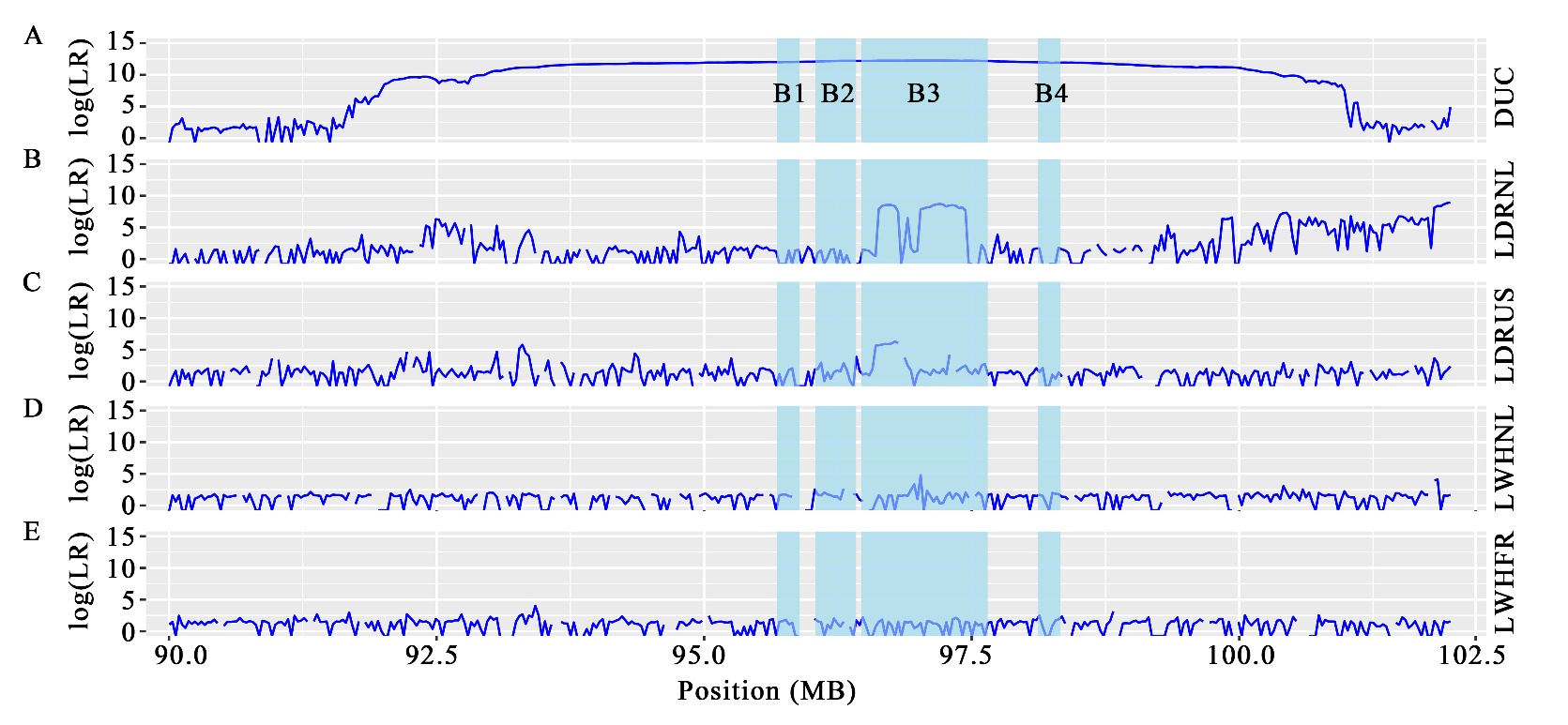
**

**Suppl. Fig. S6** VolcanoFinder result of Western commercial pigs on chr14:92 – 102 MB. Log likelihood ratio of adaptive selection on chr14:90–102MB of (A). Duroc, (B). Dutch Landrace, (C). American Landrace, (D). Dutch Large White, and E. French Large White.


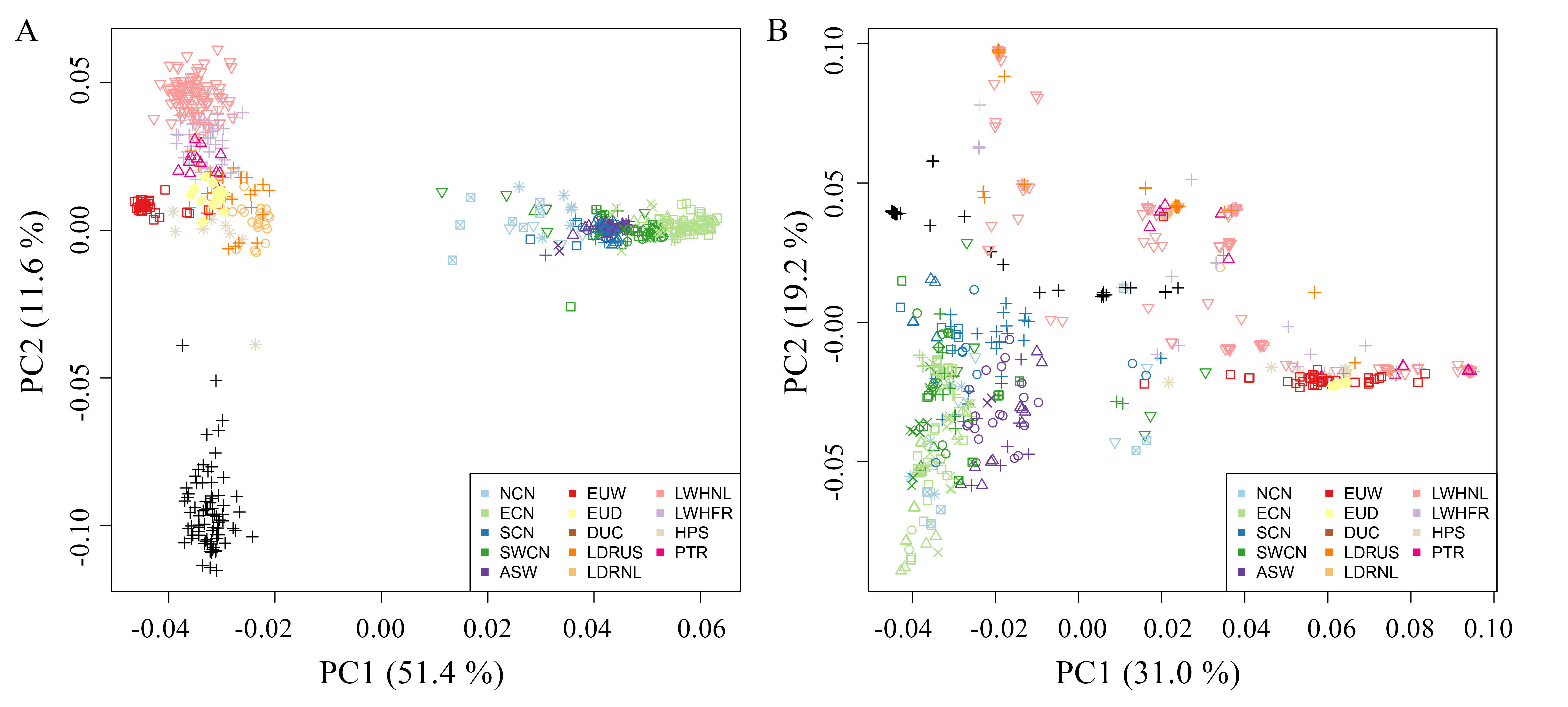


**Suppl. Fig. S7** PCA analysis on (A). Whole-genome and (B). local genome (chr14:95.68–98.33 MB).


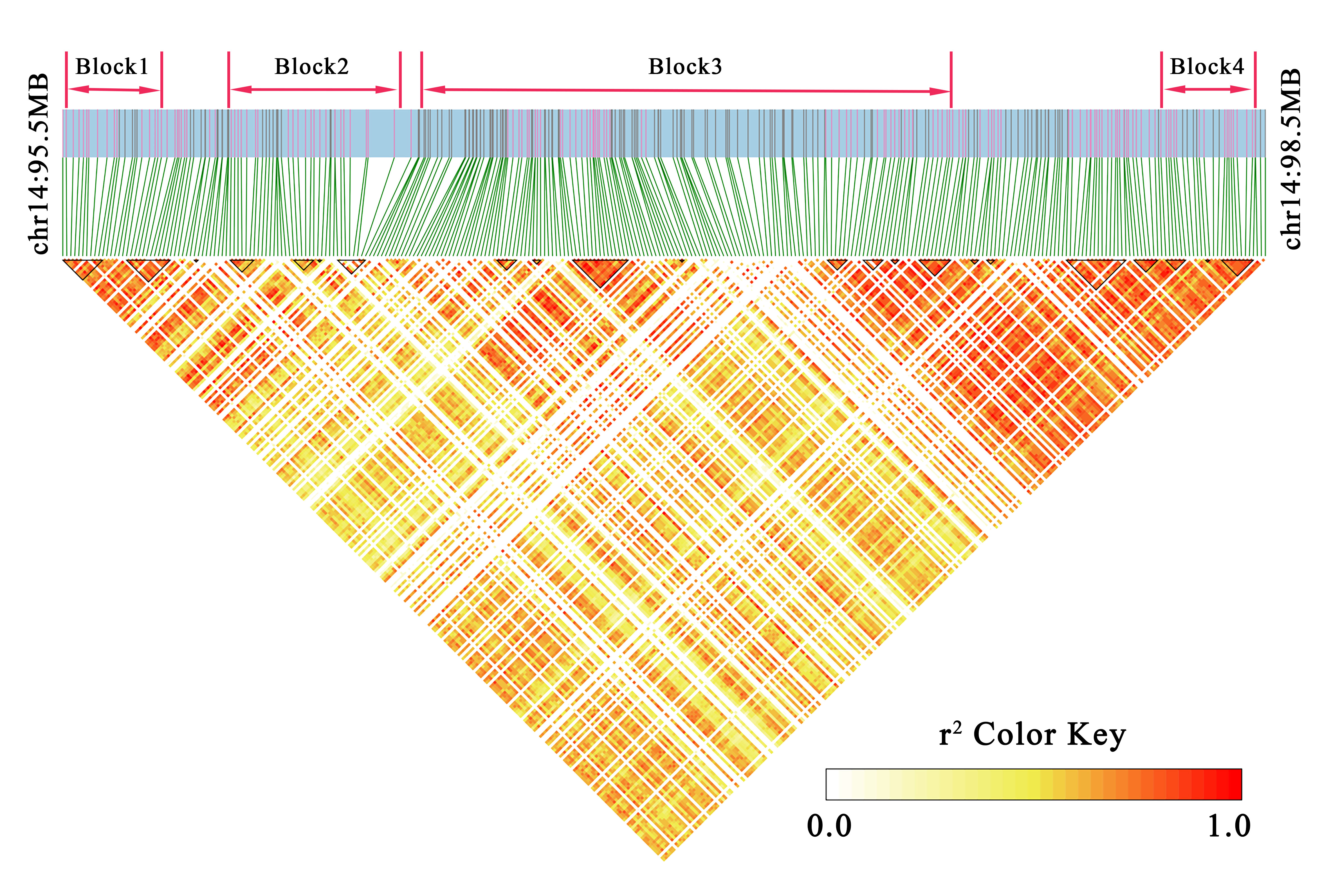


**Suppl. Fig. S8** Linkage heatmap of r^2^ in the Duroc population of chr14:95.5–98.5MB. The SNPs were pre-filtered according to LD by Plink-1.9 (--indep-pairwise 50 10 0.1). After filtering, a total of 314 SNPs were left in the heatmap.


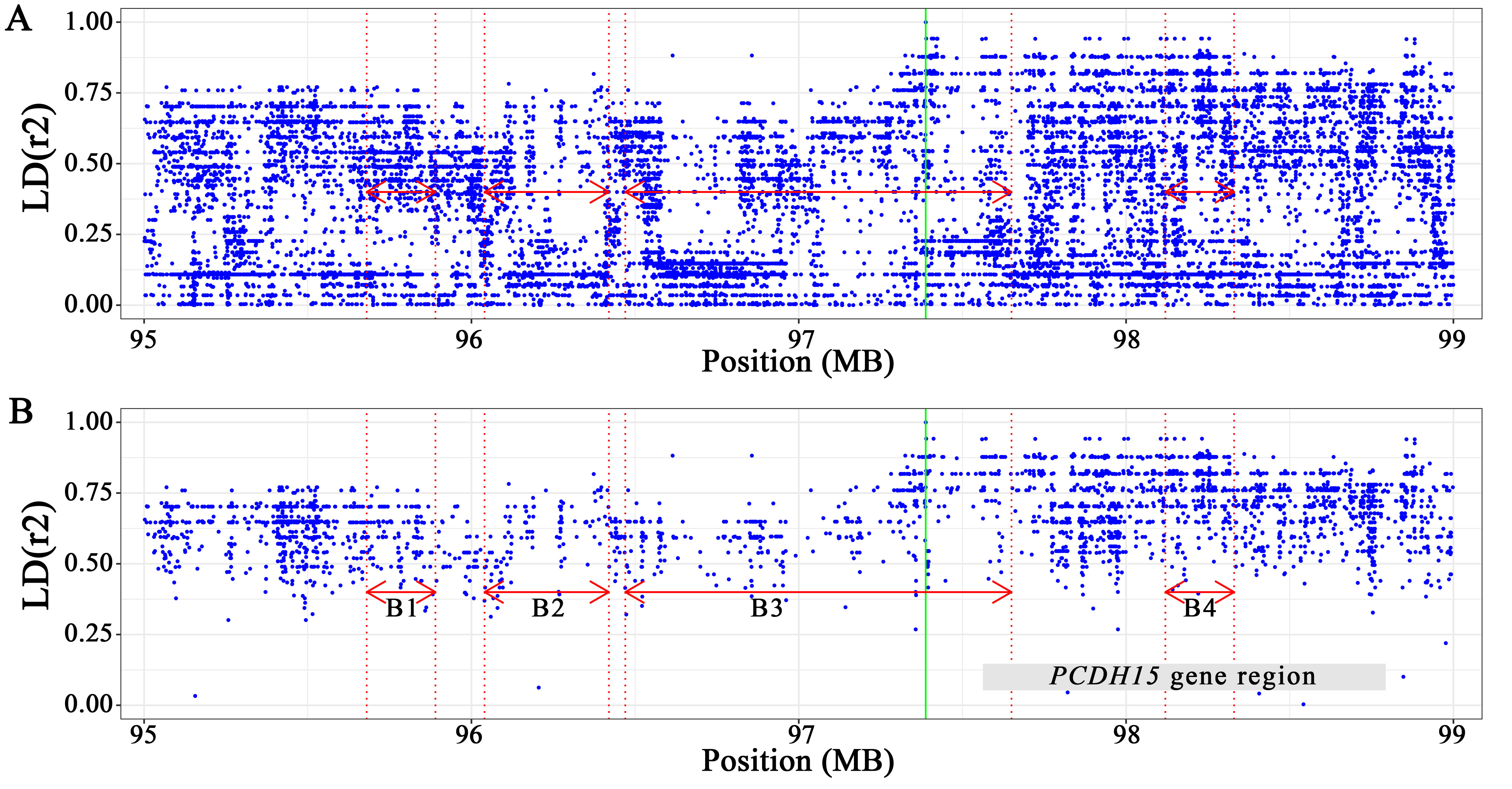


**Suppl. Fig. S9** Linkage disequilibrium (LD) level of INRA0045978 (chr14:97387849) with (A). all variants in this region, and (B). Chinese-derived variants in the Duroc population. The position of chr14:97387849 is marked by the green line.

## Reference:

Wang., L., et al. (2011). "Animal Genetic Resources in China: Pigs." Beijing, China: China Agriculture Press.
